# Elucidating the toxic effect and disease mechanisms associated with Lyso-Gb3 in Fabry disease

**DOI:** 10.1093/hmg/ddad073

**Published:** 2023-05-05

**Authors:** Valeria Nikolaenko, David G Warnock, Kevin Mills, Wendy E Heywood

**Affiliations:** Genetics & Genomic Medicine Department, Translational Mass Spectrometry Research Group, London WC1N 1EH, UK; Great Ormond Street Hospital Biomedical Research Centre, UCL Institute of Child Health, London WC1N 1EH, UK; Division of Nephrology, University of Alabama at Birmingham, Birmingham, AL 35298, USA; Genetics & Genomic Medicine Department, Translational Mass Spectrometry Research Group, London WC1N 1EH, UK; Genetics & Genomic Medicine Department, Translational Mass Spectrometry Research Group, London WC1N 1EH, UK; Great Ormond Street Hospital Biomedical Research Centre, UCL Institute of Child Health, London WC1N 1EH, UK

## Abstract

Fabry disease stems from a deficiency of alpha-galactosidase and results in the accumulation of globotriaosylceramide (Gb3). However, the production of its deacylated form globotriaosylsphingosine (lyso-Gb3) is also observed and its plasma levels have closer association with disease severity. Studies have shown that lyso-Gb3 directly affects podocytes and causes sensitisation of peripheral nociceptive neurons. However, little is understood of the mechanisms of this cytotoxicity. To study the effect on neuronal cells, we incubated SH-Sy5y cells with lyso-Gb3 at low (20 ng/mL) and high (200 ng/mL) levels, to mimic mild and classical FD serum levels. We used glucosylsphingosine as a positive control to determine specific effects of lyso-Gb3. Proteomic analyses revealed that cellular systems affected by lyso-Gb3 included cell signalling particularly protein ubiquitination and protein translation. To confirm ER/proteasome perturbations, we performed an immune enrichment of ubiquitinated proteins and demonstrated specific increased protein ubiquitination at both doses. The most ubiquitinated proteins observed included the chaperone/heat shock proteins, cytoskeletal proteins and synthesis/translation proteins. To detect proteins that interact directly with lyso-Gb3, we immobilised lyso-lipids, then incubated them with neuronal cellular extracts and identified bound proteins using mass spectrometry. Proteins that specifically bound were chaperones and included HSP90, HSP60 and the TRiC complex. In conclusion, lyso-Gb3 exposure affects pathways involved in protein translation and folding. This response is observed as increased ubiquitination and changes in signalling proteins which may explain the multiple biological processes, particularly cellular remodelling, often associated with FD.

## Graphical Abstract

**Graphical Abstract:**
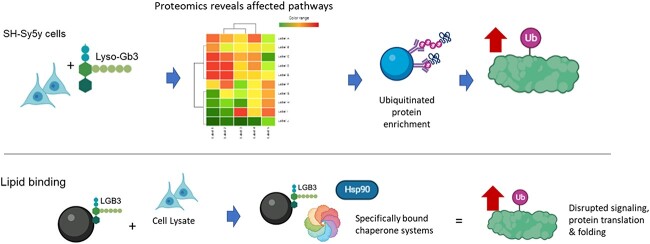

Summary of the study. Lyso-Gb3 was added to cells that were proteomically profiled. The top affected pathway was ubiquitination, which was investigated and determined to be specific to lyso-Gb3 exposure. Proteins that bind to lyso-Gb3 are associated with protein production and folding indicating disruption of protein production is a mechanism of lyso-Gb3 proteotoxicity.

## Introduction

Fabry disease (FD) is an X-linked disorder that arises from defects in the alpha-galactosidase gene which encodes for a lysosomal enzyme in the glycosphingolipid degradation pathway. This results in the accumulation of the substrate globotriaosylceramide (Gb3) in the lysosome. Typical clinical features of FD are peripheral nerve pain, progressive chronic kidney disease and hypertrophic cardiomyopathy with an increased incidence of stroke events (1). A downstream effect of Gb3 accumulation is its modification to globotriaosylsphingosine (lyso-Gb3). This is thought to occur from non-specific activity of lysosomal acid ceramidase which removes the fatty acid moiety from Gb3 (2). High levels of lyso-Gb3 can be detected in the plasma of FD patients, for which it is considered a better biomarker for disease progression than plasma Gb3 itself (3–6). Lyso-Gb3 has been used to monitor response to enzyme replacement therapy (4,7,8) and has the potential for monitoring gene therapy (9,10). However, its utility at monitoring response to treatment with Migalastat is contentious and does not correlate with improvement in clinical symptoms (11–13). Lyso-Gb3 is thought to contribute to the pathology of FD but in what manner and by how much is not fully understood. Exposure to lyso-Gb3 has been shown to cause podocyte pathology (14,15) and renal fibrosis (16), inhibit collagen synthesis in fibroblasts (17), elicit Receptor-Interacting Protein Kinase 3 (RIPK3) mediated inflammation (15) and through work carried out in our labs, to be involved in neuropathic pain (18). Several lyso-glycosphingolipids (lyso-GSLs) associated with neurodegenerative diseases have been demonstrated to affect critical oligodendrocyte functions and lysosomal function, and postulated to be due to their free amine group affecting pH in the lysosome itself (19). However, whilst this could be a common mechanism of lyso-GSL toxicity it does not explain the large differences in disease severity and phenotype that are associated with the different lyso-GSLs. For example, the biomarker lyso-GSLs psychosine and glucosylsphingosine are almost identical molecules except for the orientation of a single hydroxyl group. However, psychosine is associated with the severe neurodegenerative Krabbe disease and glucosylsphingosine is associated with Gaucher disease (GD), which has a very different clinical phenotype to Krabbe disease. Levels of lyso-Gb3 are not known to have been explored in the brain of FD patients and as it has only been found in low levels in the brain of FD mouse models, this may explain the lack of neurodegeneration observed in FD compared with the other glycosphingolipidoses (20). Nevertheless FD does affect the nervous system as peripheral nerve damage and severe bouts of pain is a hallmark of the disease and not a feature of Gaucher or Krabbe disease (17). With this in mind, we set out to evaluate the effects of lyso-Gb3 on neuronal cells using SH-Sy5y cells as a model, in the attempt to understand the specific molecular effect of lyso-Gb3 on the cell and highlight its importance as a disease biomarker in FD.

## Results

### Lyso-Gb3 exposure and its effect on the SH-Sy5y cellular proteome

The plasma range of lyso-Gb3 in untreated FD patients has been observed up to 320 ng/mL (407 nm) (21) and for glucosylsphingosine up to 250 ng/mL (542 nm) in untreated GD patients (22) but depends on the type and severity of the mutation. To test the acute effect of lyso-Gb3 toxicity on the proteome, SH-Sy5y cells were incubated with lyso-Gb3 and glucosylsphingosine at 20 ng/mL for 24 hours. This equates to 25.5 nm lyso-Gb3, typically observed in an FD-affected male, and 43.5 nm glucosylsphingosine, observed in a milder type I GD-affected patient. SH-Sy5y cells were also incubated at a very high dose of 200 ng/mL for 72 hours to explore the longer term effect at the most toxic levels expected in both diseases.

**Figure 1:**
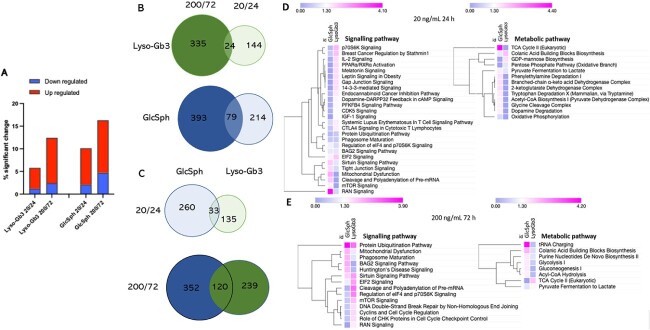
The effect of lyso-Gb3 and glucosylsphingosine (GlcSph) on the SH-Sy5y cell proteome demonstrating significant changes in protein expression. (**A**) The overall percentage of significantly altered proteins (Y-axis) from exposure to 20 and 200 ng/mL lyso-Gb3 and glucosylsphingosine (ANOVA *P* < 0.05). (**B**) Venn diagrams showing the overlap of proteins affected at the 20 and 200 ng/mL doses by each lyso-lipid (green shades pertain to lyso-Gb3, blue to glucosylsphingosine). (**C**) Venn diagrams showing the overlap between the lyso-lipids at each dose. Heat maps of significantly altered signalling and metabolic canonical pathways for (**D**) 20 and (**E**) 200 ng/mL. Ingenuity pathway analysis software was used to investigate affected pathways. Colour indicates −log (*P*-value): purple colour represents *P* > 0.05, pink colour represents *P* < 0.05; the darker the pink colour, the more significant the *P*-value.

All cell conditions were subjected to proteomic analysis and compared to a DMSO control for changes in differential protein expression and the identification of potential disease mechanisms. The positive control group of glucosylsphingosine resulted in the greatest effect on the total cellular proteome at both doses, with approximately 10% and 16% of the proteome significantly altered, respectively (Fig. 1A). Lyso-Gb3 also had a significant but slightly lower effect on the changes in cellular protein expression of 5.8% and 12.4% for the 20 and 200 ng/mL lipid concentrations, respectively. Of the proteins significantly affected in each condition, the majority (approximately 80%) showed an upregulation for the lower dose. However, the 200 ng/mL glucosylsphingosine exposure showed a slightly greater number of downregulated proteins (29% *vs* 71% upregulated proteins) than lyso-Gb3 exposure at 200 ng/mL. To explore the effect each lipid has on the proteome at both doses, we first looked at proteins that changed in common across the datasets to determine general lyso-glycosphingolipid response. When comparing low and high doses of lyso-Gb3 exposure, there was little overlap of only 24 shared protein changes (4.7%). This was also observed for glucosylsphingosine with just 79 (11.5%) protein changes shared between the two doses (Fig. 1B). This indicates that acute and longer (chronic) exposure has different effects, with different biological processes or responses occurring over time (Fig. 1D, E). We also examined the specific effects of each lipid and determined which proteins were equally affected and were in common at each dose. Interestingly, at both doses, there was little overlap between the two lipids with 33 (7.7%) proteins shared at 20 ng/mL and slightly greater 120 (16.9%) number shared at 200 ng/mL (Fig. 1C). This indicates that the mechanism of toxicity elicited by the two lipids is different and very specific, thereby explaining why the phenotypes of the two diseases are also vastly different.

To investigate what biological processes are affected by these lyso-glycosphingolipids, we also performed a pathway analysis using bioinformatic Ingenuity Pathway Analysis tool. This analysis indicated that signalling pathways were more affected by lyso-Gb3, whereas metabolic pathways were more affected by glucosylsphingosine. The most affected pathways in lyso-Gb3 at the low dose exposure were identified to be those involved in protein synthesis (p70S6K signalling that regulates transcription), folding (GDP-mannose biosynthesis that is a source of mannose for protein glycosylation) and translation (EIF2 signalling that mediates binding of tRNA to the ribosome). At the higher dose of lyso-Gb3 exposure, pre-mRNA processing and protein ubiquitination pathways were also observed to be affected (Fig. 1D, E). Although protein ubiquitination became more affected at the high dose of glucosylsphingosine, it was the TCA cycle, RAs-related nuclear protein (RAN) signalling, which concerns nucleocytoplasmic transport, and mitochondrial dysfunction that were the pathways most affected. Many pathways involved in energy metabolism were seen to be affected by the lower dose of glucosylsphingosine (Fig. 1D). Lyso-Gb3 did appear to affect the TCA cycle but only at the higher dose (Fig. 1E). These data indicate that lyso-Gb3 exposure elicits a specific and more potent effect on protein synthesis and folding, whereas glucosylsphingosine exerts a more potent effect on energy metabolism.

### Protein ubiquitination and lyso-glycosphingolipid exposure: a potential disease mechanism caused by lyso-Gb3 as a disruptor of the protein synthesis/folding machinery of the cell

**Figure 2:**
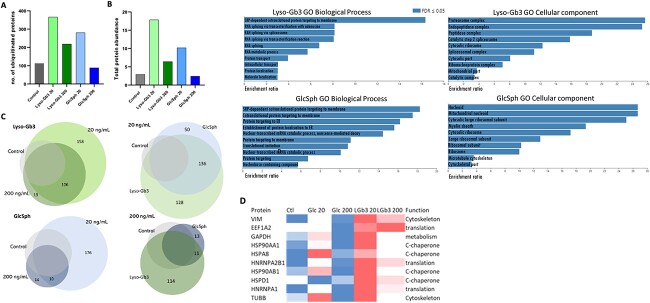
Ubiquitination of lyso-glycosphingolipid exposed proteins. (**A**) The number of proteins in each condition that bound to anti-mono and polyubiquitin chain antibody. (**B**) The estimated total ubiquitinated protein amount by sum of all protein abundance. (**C**) Venn diagrams showing the overlap of ubiquitinated proteins identified for each condition. (**D**) Heatmap of the top 10 most ubiquitinated proteins in both lyso-Gb3 conditions. Red colour indicates greater values, blue—lower, white—values similar to those in control. (**E**) GO analysis indicating the enriched biological functions and cellular components that may be affected by each lyso-lipid exposure.

To explore further the role of lyso-Gb3 and its effect on protein folding and increased targeting of proteins to the proteasome, we investigated the most significantly affected pathway of ‘protein ubiquitination’ in our cell model. Lysate from cells that had been exposed to the different doses of each lyso-GSL was incubated with an anti-poly and mono ubiquitin chain antibody, both to immuno-enrich and isolate proteins containing the ubiquitination modification. Proteomic analyses of ubiquitinated proteins demonstrated that the 20 ng/mL dose resulted in the greatest effect on ubiquitination, with over 200 ubiquitinated proteins specifically detected for lyso-Gb3 and 168 proteins for glucosylsphingosine exposure (Fig. 2A) (Supplementary Material, Tables S1 and S2). A smaller number of proteins were detected for the 200 ng/mL lyso-Gb3 dose but interestingly the glucosylsphingosine group did not show any change in the level of ubiquitination at this dose. When totalling the abundance of all ubiquitinated proteins identified at the 20 ng/mL dose, we see lyso-Gb3 exposure has a total ubiquitination almost double that of glucosylsphingosine (Fig. 2B). Ubiquitination was notably lower in the 200 ng/mL exposed cells but still increased in lyso-Gb3 exposure compared to the control group and unaffected glucosylsphingosine group. When exploring the degree of overlap between the doses and lyso-lipids we see the two doses of lyso-Gb3 almost completely overlap, thereby eliciting similar ubiquitination effects except with less proteins observed for the 200 ng/mL dose (Fig. 2C). The 200 ng/mL glucosylsphingosine dose did not show an overall increase in ubiquitination and exhibited a much smaller overlap (5%) with the 20 ng/mL dose. This indicates that the low dose of both lipids elicits a sharp increase in ubiquitination but more so for lyso-Gb3. The same is apparent when comparing the ubiquitinated proteins between lyso-lipid exposures that shows a greater number of specific proteins in the lyso-Gb3 exposure at both doses (Fig. 2D).

To identify what protein classes were being ubiquitinated and the pathways these proteins were involved in, we subjected the set of ubiquitinated proteins identified for each group to Gene Ontology analysis (Fig. 2E). Biological processes more unique to lyso-Gb3 exposure were found to be those involved in mRNA metabolism, whereas glucosylsphingosine exposure indicates changes in proteins involved in trafficking or transport to the endoplasmic reticulum (ER). Cellular components that were more specific to ubiquitinated proteins from lyso-Gb3 exposure were the proteosome and endopeptidase complex and for glucosylsphingosine they were mitochondrial nucleoid and myelin sheath. Both lyso-GSLs appeared to affect ribosomal function. Therefore, both lyso-glycosphingolipids affect protein synthesis and although with some overlap, but weighted to slightly different mechanisms. The proteins most ubiquitinated by lyso-Gb3 quantitatively are listed in the heatmap table in Figure 2 and include the cytoskeletal protein vimentin, EE1A2, which is involved in protein translation, heterogeneous ribonucleoproteins involved in RNA transport and cytosolic chaperones.

### Lyso-Gb3 specifically binds and causes ubiquitination of the cell’s chaperone systems

**Figure 3:**
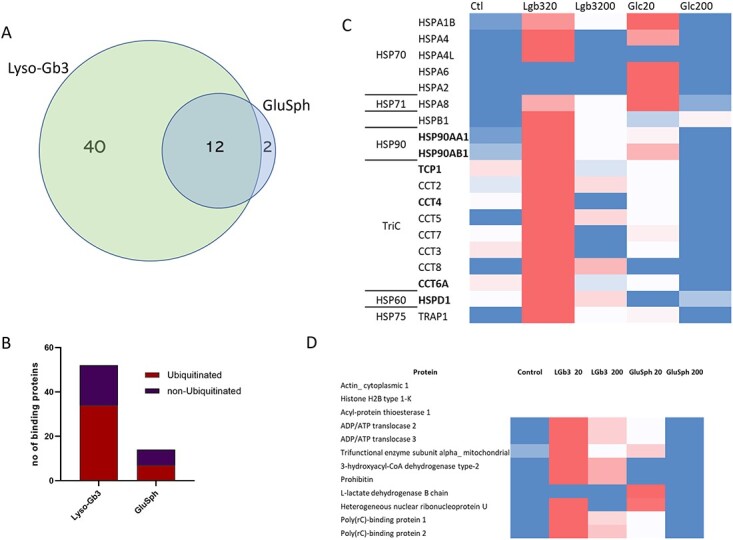
Protein binding to lyso-Gb3 and glucosylsphingosine (GlcSph). (**A**) Venn diagram showing the majority of proteins bind to lyso-Gb3. (**B**) The number of proteins that bound to the lyso-glycosphingolipid that were also found to be ubiquitinated. (**C**) Heatmap of the chaperone proteins that are highly ubiquitinated and bind to lyso-Gb3, colour as shown in Figure 2(D). (**D**) Level of ubiquitination of proteins that bound to both lyso-GSLs, colour as previously. Note: Actin and H2B bound but were not ubiquitinated.

To explore which proteins have a high affinity for lyso-Gb3, we attempted to look for proteins that specifically interact with lyso-Gb3. This was performed by immobilizing lyso-Gb3 via covalent binding of its free amine group with an epoxy group on the magnetic beads and incubating with an SH-Sy5y cell lysate. Blank beads were used to detect non-specific binding and as a positive control, glucosylsphingosine was also bound to the beads and subjected to the same analyses. Identified proteins are listed in Supplementary Material, Table S3. Figure 3(A) shows the proteomic analyses of these proteins and the number of proteins with a high affinity and binding capacity to each lyso-GSL. The Venn diagram demonstrates that lyso-Gb3 binds far more proteins than glucosylsphingosine and those that do bind to glucosylsphingosine also bind to lyso-Gb3. We then cross referenced the binding proteins with the ubiquitinated protein analysis to see if lyso-Gb3 binding of certain proteins, or protein complexes, may be causing these proteins to become ubiquitinated. Figure 3B shows the proportion of lyso-lipid-bound proteins that become ubiquitinated. The proportion of ubiquitinated proteins that also bound lyso-Gb3 was greater (~65%) than that observed for glucosylsphingosine (50%). This indicates that the increased ubiquitination observed likely comes from direct binding by lyso-Gb3, which could potentially render the protein inert or more difficult to ‘fold’ by the chaperone system of the cell. When checking the proteins that bind and are highly ubiquitinated, we observed many of these were cytosolic chaperone components including HSP90AA1 and HSP90AB1, TRAP1 a mitochondrial chaperone, 3 components of the T-complex protein Ring Complex (TRiC) and HSPD1, a component of Hsp60. Figure 3C shows a heatmap table of the chaperone complex proteins that had increased ubiquitination and those that directly bound are indicated in bold. Both key components for the HSP90 chaperone class bound and had increased ubiquitination. These were some of the most abundant ubiquitinated proteins observed. Although only three components of the TRiC complex bound to lyso-Gb3 other components of this complex were ubiquitinated and indicate that lyso-Gb3 likely disrupts the entire complex through its binding to the TCP1, CCT4 and CCT6A subunits. HSP90AA1, CCT3 and CCT4 of the TriC complex were detected in the proteomic profiling analysis and were only observed to be significantly upregulated in the 200 ng/mL lyso-Gb3 exposure and not the 20 ng/mL. This indicates at 20 ng/mL the protein is likely less functional due to its increased ubiquitination without increased expression. At 200 ng/mL, which was incubated for 72 hours, the protein expression is greater likely due to the need to compensate for the ubiquitinated/lyso-lipid damaged chaperones. This is the likely scenario for why the proteome is more affected at 200 ng/mL for 72 hours, as it is responding to the proteotoxic events that occur initially.

Some proteins that had shared binding between the two lyso-glycosphingolipids also demonstrated an increase in ubiquitination and are shown in Figure 3D. Not all binding proteins were ubiquitinated which included actin, histone H2B and Acyl-protein thioesterase 1 (LYPLA1). Many of the proteins that bound to both lyso-lipids were mitochondrial (associated with proton transport or fatty acid metabolism) or related to protein translation. Most shared proteins had greater ubiquitination in lyso-Gb3 exposed cells apart from lactate dehydrogenase.

## Discussion

Proteomic analysis of the SH-Sy5y cell proteome after exposure to lyso-Gb3 or glucosylsphingosine showed that a higher dose of 200 ng/mL for 72 hours elicited a greater change in protein expression than a lower 20 ng/mL dose for a shorter period (Fig. 1A). However, protein ubiquitination which was a key affected pathway from the proteomics analysis seems to have a greater effect at the lower dose for both lyso-GSLs (Fig. 2A). This indicates that there are differences between an acute and chronic responses to lyso-GSL exposure. However, the ubiquitination level normalizes or adapts in glucosylsphingosine exposure but for lyso-Gb3 it remains increased indicating a more specific effect of lyso-Gb3 on protein folding and proteasomal targeting by ubiquitination. Three proteins involved in the protein ubiquitination process, such as ubiquitin-like modifier-activating enzyme 1 (UBA1), a ubiquitin-activating enzyme, ubiquitin-conjugating enzymes E2 N and E2 D3 (UBE2N and UNE2D3), were themselves observed to be increased in ubiquitination in the lyso-Gb3 exposure. Reduction of these proteins would further affect protein ubiquitination and their targeting for proteasomal degradation. We observed that proteasome 20S core subunit alpha type-2 (PSMA2) and 26S proteasome non-ATPase regulatory subunit 1 (PSMD1) bound to lyso-Gb3. If the proteosome is disrupted from lyso-Gb3 this could explain the increase in ubiquitinated proteins. The cell exposed proteomic analysis demonstrated an upregulation of PSMD1 and other proteosome proteins as well as UBE2N only in the 200 ng/mL longer 72-hour groups for both glucosylsphingosine and lyso-Gb3 exposure. This indicates that this increased expression at 200 ng/mL cannot be attributed to the disruption of the proteosome by lyso-Gb3 as glucosylsphingosine also induced this effect. The most significant of the proteins affected by ubiquitination included the major chaperone proteins of the ER calnexin/calreticulin and protein disulfide-isomerase A3 (PDIA3), which are particularly involved in the correct folding of glycoproteins and disulphide bond formation (Supplementary Material, Table S1). Calnexin and calreticulin specifically recognize carbohydrate moieties and hydrophobic areas of proteins and hence a theory could involve misrecognition of lyso-Gb3 as an unfolded protein. However, calnexin and calreticulin specifically recognize terminal glucose residues (23) and not galactose as contained in lyso-Gb3 so this theory will need to be confirmed. Interestingly, when cross comparing the ubiquitinated protein capture data sets with proteins affected in the overall proteome, only ~ 4.2% of these proteins were observed to be altered in the proteomics analysis. This could be explained as the proteins whose ubiquitination is the most affected by lyso-Gb3 are chaperones that are critical for the correct protein folding, and the downstream effect on total protein expression could be significant despite an overall small percentage of affected proteins. Furthermore, other affected biological processes included protein translation and RNA metabolism, which could be a separate and additional disease mechanism or indeed be caused by the proteins critical for protein translation/regulation themselves being affected by the targeting to the proteasome for premature degradation.

To see if a direct interaction of lyso-Gb3 could cause an event that would result in an exaggerated protein ubiquitination response, we bound both lyso-GSLs to beads and incubated with a cell lysate. Lyso-Gb3 ‘pulled out’ a greater number of proteins than glucosylsphingosine, and 65% of these were observed to be ubiquitinated, compared to 50% for glucosylsphingosine (Fig. 3B). The most abundant proteins that were ubiquitinated appeared to be associated with cytosolic and ER chaperone activity (Fig. 3C). Of particular interest were the subunits alpha and beta of heat shock protein HSP90 that both bound lyso-Gb3 and had high levels of protein ubiquitination. These two independent experiments confirm a highly likely relationship between an affinity for lyso-Gb3 and a resulting targeting to the proteasome for destruction because of an inability to fold these proteins correctly. Total expression of HSP90 alpha was observed to be increased by approximately sixfold only at 200 ng/mL/72 hours and indicates the upregulation of protein synthesis in response to the large proportion of the protein being targeted for proteasomal degradation. HSP90 is an abundant cellular chaperone that mediates folding of many ‘client proteins’ and is involved in the regulation of cell division, proliferation and apoptosis. HSP90 also activates numerous factors involved in inflammation, fibrosis and hypoxia (24–26) and overall is considered a master regulator of proteostasis. This indicates lyso-Gb3 may cause direct disruption of the chaperone system both in the ER and the cytosol, resulting in an increase in ubiquitination from a proteotoxic response likely through HIF1 activation (27). This leads to increased production of HSP90 to counter the disruptive effect of lyso-Gb3. HSP90 has many co-chaperones (including HSP70) and client proteins many of which are transcription factors and signalling kinases (see www.picard.ch/downloads (28,29)). Disruption of HSP90 function may explain the greater changes in the cell proteome at 200 ng/mL/72 hours and the downstream effect on signalling pathways that we observe only in lyso-Gb3 exposed cells in the 200 ng/mL 72-hour group (Fig. 1E). Many of these disrupted signalling pathways likely lead to tissue specific pathological processes observed in FD, such as previously observed Notch1 activation in FD patient kidney biopsies with lyso-Gb3 exposure in podocytes (30) and RIPK3 signalling (15). A limitation of this study was the cell type used as we wished to study the effect of lyso-Gb3 on neuronal cells to provide insight into pain often observed with FD patients. Thus, we only describe the disruption of HSP90 chaperone system in SH-Sy5y cells but if this effect is also observed in other tissues such as podocytes, endothelial, and cardiac cells, it could account for some of the pathological features of FD. BiP (HSPA5/GRP78) is the key ER chaperone that increases with ER-associated degradation (ERAD) and has been observed to be increased in sensory neurons of alpha galactosidase deficient mice (31). We were not able to detect BiP in the proteomic profiling analysis but observed an increased ubiquitination of BiP at both lyso-Gb3 exposures (Supplementary Material, Table S2) as well as calnexin, calreticulin and PDIA3. ER stress and the unfolded protein response (UPR) have been described previously as a potential disease mechanism that occurs in FD (31,32). However, it is thought to occur from misfolding of the mutant alpha galactosidase. Our findings also indicate that the UPR could be triggered in FD from disruption of chaperone activity caused by action of lyso-Gb3. This likely has a downstream effect on the ER protein folding thereby also triggering ERAD.

Other interesting proteins that indicated a susceptibility to lyso-Gb3 were the three subunits of the TriC complex aka chaperonin containing TCP-1 (CCT). Higher levels of ubiquitination for all of the TRiC complex subunits were observed indicating the entire complex is affected by lyso-Gb3 exposure. The TRiC/CCT complex facilitates the folding of cytoskeletal actins and tubulins which comprise ~ 10% of the cellular proteome (33). TRiC is thought to play a role in protection from protein aggregation by modulating amyloidosis (34), and downregulated TRiC can impair autophagy due to compromised folding of actin/tubulin needed for autophagy functions (35). In relevance to neuronal cells, TRiC contributes to axonal function as overexpression of TRiC enhances retrograde axonal transport by modulating tau phosphorylation (36). Interestingly, mutations in the TRiC subunits CCT4 and CCT5 are associated with hereditary sensory neuropathies (37,38) implicating that dysfunction of TRiC in axons from lyso-Gb3 exposure could be a contributor to the peripheral neuropathy observed in FD. Fabry disease is known to be a very heterogeneous disease affecting different tissues and organs in different ways. Many of the proteins affected by lyso-Gb3 in this work also have heterogeneous expression such as the proteosome (39) and chaperone systems (40); this variability could contribute to the heterogeneity observed in FD.

In summary, this study has identified and confirmed specific mechanisms that affect chaperone function in lyso-Gb3-exposed cells. A limitation of this study is that SH-Sy5y cells used are a cancer cell line in a constant proliferative state, which could show effects more related to cell proliferation. However, many of the observations do relate to what could be causative of the FD phenotype such as altered signalling pathways. Further studies are needed to focus on and elucidate the roles of HSP90 and TRiC on cell types affected in FD such as podocytes, cardiomyocytes and endothelial cells as well as the long-term effects of lyso-Gb3 exposure on these systems. Another limitation is that immobilising the lyso-lipids to epoxy beads may obscure the free amine group as this is the likely linker to the epoxy group. However, the effects on the cell proteome and associated disease phenotypes of the two lyso-GSLs are so different we suspect that the differences are largely caused by the different sugar moieties and not just the common amine group that both lipids have. Hence, why a comparison with glucosylsphingosine was considered the best way to look for specific effects of lyso-Gb3. Understanding the toxic mechanism of lyso-Gb3 could open a field of clinical research that could identify treatment to target lyso-Gb3 and augment alpha-galactosidase replacement or at least bring lyso-Gb3 under control so replacement therapy can establish. One other clinical aspect of this work is that it highlights how relevant lyso-Gb3 is to disease pathology in FD and its importance as a biomarker. Its use to monitor treatment response is valuable and observations where clinical phenotype improves with no change in lyso-Gb3 should be explored further. This lack of change could be due to a tissue-specific effect of treatment where plasma lyso-Gb3 may not represent lyso-Gb3 in all organs.

### Data availability

Datasets produced in this study are available to download at https://figshare.com/s/9da0bc4009da7f7058a4.

## Materials and Methods

### Cell culture

SH-Sy5y cell line was purchased from the European Collection of Cell Cultures (Public Health England, UK). SH-Sy5y cells used in this study were between passages 8 and 15. Cells were incubated in DMEM/F12 (1:1) supplemented with 10% human serum (Sigma, UK) due to high level of endogenous glucosylsphingosine present in standard 10% FBS supplemented culture medium. Cell viability was assessed using AlamarBlue™ HS cell viability reagent (Invitrogen, Thermo Fisher) as per manufacturer’s instructions. Cells were plated in six-well plates at the density of 3 × 10^5^ cells/well and treated with lyso-Gb3/glucosylsphingosine (Avanti lipids) dissolved in DMSO or vehicle for 24 hours at the 20 ng/mL dose and 72 hours at the 200 ng/mL dose. Cells were harvested using trypsin and centrifuged at 500 × *g* for 5 min and further washed with PBS.

### Label-free proteomics and deep proteomic phenotyping

Pelleted cells were lysed in PBS by three freeze–thaw cycles using 37 °C bath and dry ice. Cell lysate was ice-cold acetone precipitated at a volume of 2:3 and left at −20 °C for 4 hours, then centrifuged to pellet the precipitated protein. The supernatant was removed and the protein pellet air dried. Precipitated protein was digested as described previously (19). Briefly, protein was solubilized by the addition of 20 μL of 100 mm Tris buffer (pH 7.8) containing 6 m urea, 2 m thiourea and 2% (w/v) amidosulfobetain-14. Disulphide bridges in proteins were broken by the addition of 45 μg of dithioerythritol (DTE) prepared fresh at 30 mg/mL and left on the shaker for 60 minutes. Carbamidomethylation of cysteines was performed by the addition of 108 μg of iodoacetamide (IAA) prepared fresh at 36 mg/mL and samples incubated in the dark for 45 minutes. DTE and IAA solutions were made in 100 mm Tris buffer (pH 7.8). Urea concentration in the samples was reduced to below 1 m by the addition of 160 μL of Milli-Q water and 1 μg of trypsin Gold (Promega, Germany) was added to each sample and incubated at 37 °C for 16 hours. Peptides were subjected to high pH, low pH fractionation as previously described using Isolute 100 mg C18 cartridges (Biotage, Sweden) (21) and eluted into five fractions at 6–50% ACN. Peptide eluents were dried by centrifugal evaporation and resuspended at a concentration of 300 ng/μL in 3% ACN, 0.1% FA.

Ultra-definition mass spectrometry (UDMS^E^) analysis was performed as previously described (21). Briefly, 0.3 μg of peptides were loaded onto a Waters NanoAquity liquid chromatography (LC) system and separated over 60 minutes on a 75 μm × 150 mm, 1.7 μm Peptide BEH C18 analytical column (Waters, UK), then injected into a Synapt-G2-Si mass spectrometer with ion mobility separation (IMS) (Waters, UK).

Raw data were imported to Progenesis QI version 4.1 (Waters, UK) and each fraction was processed separately before all five fractions were combined into one experiment. MS/MS spectra in the MS raw data files were searched against the UniProt most recent published human reference proteome database with manual addition of porcine trypsin (P00761). Enzyme digestion was set to trypsin, maximum missed cleavage number was set to 3. Cysteine carbamidomethylation was set as a fixed modification and methionine oxidation, protein N-terminal acetylation, glutamine deamidation and protein N-terminal pyrrolidone carboxylation were set as variable modifications. The false discovery rate (FDR) cut-off was set at 1% at both peptide and protein level. Protein quantification was performed using unique peptides only. The resulting protein identifications and quantitative data were exported to Excel (Microsoft) for further analysis.

### LC-MS/MS analysis of lyso-Gb3

To confirm entry of lyso-lipids into the cells, cell media and cell lysate were subjected to LC-MS/MS analyses using in-house methods with minor modifications as previously described (41).

### The immobilisation and the identification of lyso-glycosphingolipid interacting proteins

Glucosylsphingosine and lyso-Gb3 standards in MeOH were evaporated by centrifugation. Thereafter, glycosphingolipids were reconstituted in 150 mM phosphate-buffered saline (PBS, pH 7.4) to a final concentration of 1 mg/mL. Five milligrams of Dynabeads® M-270 epoxy (Invitrogen, UK) were resuspended in 2 mL of dimethylformamide organic buffer, vortexed and aliquoted into seven vials (blank, sample and a duplicate) at the concentration of 3.3 × 10^8^ beads per vial. Buffer was carefully removed from the beads using a magnet. Beads were washed with 1 mL of 150 mm PBS (pH 7.4) and then resuspended in 0.1 mL of 100 mm sodium phosphate buffer (pH 7.4) and vortexed. One hundred microlitres of 1 mg/mL lyso-Gb3, glucosylsphingosine or PBS (blank) was added to each aliquot of beads. To enhance binding of lyso-glycosphingolipids to the beads, 0.1 mL of an ammonium sulphate buffer (1 m final concentration) was also added. The mixture was incubated at room temperature while mixing on a rotor for 24 hours. Following incubation, the beads with bound lyso-GSLs were placed on a magnet and supernatants removed. The beads were washed with 0.5 mL of 150 mm PBS (pH 7.4) containing 2.5 mg/mL of blocking agent horse myoglobin to reduce nonspecific binding. Beads were further washed in PBS. Lyso-GSL bound beads were resuspended in 0.1 mL of the 100 mm sodium phosphate buffer (pH 7.4), 0.035 mg/mL protein from SH-SY5Y cell lysate diluted in 150 mm PBS (pH 7.4) added and incubated at room temperature for 1 hour while mixing on the rotor. Supernatants were removed and beads washed with 0.5 mL of 150 mm PBS containing 0.1% Triton-100 (pH 7.4) for 15 min. Triton wash was removed and beads were washed with 150 mm PBS (pH 7.4) twice. Protein–glycolipid-bound partners were eluted from the beads by the addition of 40 μL of 100 mm Tris (pH 7.8), containing 6 m urea, 2 m thiourea, 0.2% ABS-14 (w/v), subjected to in-solution digestion without fractionation and LC–MS/MS proteomics analysis (21). Data were imported to ProteinLynx Global SERVER v3 (Waters, UK), and protein identification was performed using UniProt’s most recent published human reference proteome FASTA database (https://www.uniprot.org/proteomes/UP000005640) with manual addition of porcine trypsin (P00761). Protein quantitative data were exported to Excel (Microsoft) for further analysis. Proteins identified were considered to interact with a glycosphingolipid if they were not detected in the blank—those found to interact with the beads without glycosphingolipid bound to the beads.

### Ubiquitinated protein analysis

Mouse IgG1 antibody that recognizes mono and polyubiquitin chains (Clone FK2, Sigma, UK) on proteins and not free ubiquitin was diluted in 200 μL of PBS containing 0.02% of Tween 20 and coupled to protein G magnetic beads (Dynabeads®, Invitrogen, Sigma) at 4 μg antibody/0.5 mg beads overnight while on a rotor at 4 °C. Following this, the bead–antibody complex was placed on a magnet and the supernatant carefully removed. Then the bead–antibody complex was washed twice with 200 μL of PBS-Tween 20, and the bead–antibody complex was incubated with 250 μL horse myoglobin (2.5 mg/mL) for 15 min at room temperature while shaking to reduce nonspecific binding. Horse myoglobin was washed off twice with 200 μL of PBS-Tween 20. The bead–antibody complex was incubated for 1 hour at room temperature on the rotor with SH-SY5Y cell lysates prepared as described above. The bead–antibody–antigen complex was washed with 200 μL of PBS-Tween 20 twice, following which the antibody–antigen complex was eluted from the beads with 40 μL of digest buffer containing 100 mm Tris (pH 7.8), 6 m urea, 2 m thiourea, 2% (w/v) ASB-14 while rotating for 2 min. Supernatants containing the antibody–antigen complex were subject to proteomic analysis as previously described (20). Identification of proteins present in the FK2 antibody immunocapture was performed as described above with the only difference that mouse immunoglobulin gamma-1 chain C (P01868) from the commercial antibody was also manually added to the databank at a concentration of a 4 μg/sample. Relative protein quantitation was achieved based on the amount of mouse immunoglobulin gamma-1 chain C peptide added to beads. Data were exported to Microsoft Excel for further analysis.

## Supplementary Material

supplementary_data_revised_ddad073

## References

[1] Mehta, A.B. and Winchester, B. (2022) Lysosomal Storage Disorders: A Practical Guide. Wiley-Blackwell, Hoboken, NJ.

[2] Ferraz, M.J., Marques, A.R., Appelman, M.D., Verhoek, M., Strijland, A., Mirzaian, M., Scheij, S., Ouairy, C.M., Lahav, D., Wisse, P. et al. (2016) Lysosomal glycosphingolipid catabolism by acid ceramidase: formation of glycosphingoid bases during deficiency of glycosidases. FEBS Lett., 590, 716–725. 26898341 10.1002/1873-3468.12104

[3] Nowak, A., Mechtler, T.P., Desnick, R.J. and Kasper, D.C. (2017) Plasma LysoGb3: a useful biomarker for the diagnosis and treatment of Fabry disease heterozygotes. Mol. Genet. Metab., 120, 57–61. 27773586 10.1016/j.ymgme.2016.10.006

[4] Nowak, A., Mechtler, T.P., Hornemann, T., Gawinecka, J., Theswet, E., Hilz, M.J. and Kasper, D.C. (2018) Genotype, phenotype and disease severity reflected by serum LysoGb3 levels in patients with Fabry disease. Mol. Genet. Metab., 123, 148–153. 28728877 10.1016/j.ymgme.2017.07.002

[5] Young, E., Mills, K., Morris, P., Vellodi, A., Lee, P., Waldek, S. and Winchester, B. (2005) Is globotriaosylceramide a useful biomarker in Fabry disease? Acta Paediatr., 94, 51–54 discussion 37–58. 10.1111/j.1651-2227.2005.tb02112.x 15895713

[6] Togawa, T., Kawashima, I., Kodama, T., Tsukimura, T., Suzuki, T., Fukushige, T., Kanekura, T. and Sakuraba, H. (2010) Tissue and plasma globotriaosylsphingosine could be a biomarker for assessing enzyme replacement therapy for Fabry disease. Biochem. Biophys. Res. Commun., 399, 716–720. 20692233 10.1016/j.bbrc.2010.08.006

[7] van Breemen, M.J., Rombach, S.M., Dekker, N., Poorthuis, B.J., Linthorst, G.E., Zwinderman, A.H., Breunig, F., Wanner, C., Aerts, J.M. and Hollak, C.E. (2011) Reduction of elevated plasma globotriaosylsphingosine in patients with classic Fabry disease following enzyme replacement therapy. Biochim. Biophys. Acta, 1812, 70–76. 20851180 10.1016/j.bbadis.2010.09.007

[8] Sakuraba, H., Togawa, T., Tsukimura, T. and Kato, H. (2018) Plasma lyso-Gb3: a biomarker for monitoring fabry patients during enzyme replacement therapy. Clin. Exp. Nephrol., 22, 843–849. 29288396 10.1007/s10157-017-1525-3

[9] Yasuda, M., Huston, M.W., Pagant, S., Gan, L., St Martin, S., Sproul, S., Richards, D., Ballaron, S., Hettini, K., Ledeboer, A. et al. (2020) AAV2/6 gene therapy in a murine model of Fabry disease results in supraphysiological enzyme activity and effective substrate reduction. Mol. Ther. Methods Clin. Dev., 18, 607–619. 32775495 10.1016/j.omtm.2020.07.002 PMC7396970

[10] Jeyakumar, J.M., Kia, A., Tam, L.C.S., McIntosh, J., Spiewak, J., Mills, K., Heywood, W., Chisari, E., Castaldo, N., Verhoef, D. et al. (2023) Preclinical evaluation of FLT190, a liver-directed AAV gene therapy for Fabry disease. Gene. Ther., 11, a034017. in press. 10.1038/s41434-022-00381-y. PMC10284695 36631545

[11] Bichet, D.G., Aerts, J.M., Auray-Blais, C., Maruyama, H., Mehta, A.B., Skuban, N., Krusinska, E. and Schiffmann, R. (2021) Assessment of plasma lyso-Gb(3) for clinical monitoring of treatment response in migalastat-treated patients with Fabry disease. Gene. Med., 23, 192–201. 10.1038/s41436-020-00968-z PMC7790748 32994552

[12] Lenders, M., Nordbeck, P., Kurschat, C., Eveslage, M., Karabul, N., Kaufeld, J., Hennermann, J.B., Patten, M., Cybulla, M., Muntze, J. et al. (2022) Treatment of Fabry disease management with migalastat-outcome from a prospective 24 months observational multicenter study (FAMOUS). Eur. Heart J. Cardiovasc. Pharmacother., 8, 272–281. 35512362 10.1093/ehjcvp/pvab025

[13] Young-Gqamana, B., Brignol, N., Chang, H.H., Khanna, R., Soska, R., Fuller, M., Sitaraman, S.A., Germain, D.P., Giugliani, R., Hughes, D.A. et al. (2013) Migalastat HCl reduces globotriaosylsphingosine (lyso-Gb3) in Fabry transgenic mice and in the plasma of Fabry patients. PLoS One, 8, e57631. 23472096 10.1371/journal.pone.0057631 PMC3589404

[14] Sanchez-Nino, M.D., Sanz, A.B., Carrasco, S., Saleem, M.A., Mathieson, P.W., Valdivielso, J.M., Ruiz-Ortega, M., Egido, J. and Ortiz, A. (2011) Globotriaosylsphingosine actions on human glomerular podocytes: implications for Fabry nephropathy. Nephrol. Dialysis Transplant., 26, 1797–1802. 10.1093/ndt/gfq306 20504837

[15] Kim, S.Y., Park, S., Lee, S.W., Lee, J.H., Lee, E.S., Kim, M., Kim, Y., Kang, J.S., Chung, C.H., Moon, J.S. and Lee, E.Y. (2021) RIPK3 contributes to Lyso-Gb3-induced podocyte death. Cell, 10, 245. 10.3390/cells10020245 PMC7911493 33513913

[16] Jeon, Y.J., Jung, N., Park, J.W., Park, H.Y. and Jung, S.C. (2015) Epithelial-mesenchymal transition in kidney tubular epithelial cells induced by globotriaosylsphingosine and globotriaosylceramide. PLoS One, 10, e0136442. 26291612 10.1371/journal.pone.0136442 PMC4546309

[17] Choi, J.Y., Shin, M.Y., Suh, S.H. and Park, S. (2015) Lyso-globotriaosylceramide downregulates KCa3.1 channel expression to inhibit collagen synthesis in fibroblasts. Biochem. Biophys. Res. Commun., 468, 883–888. 26592662 10.1016/j.bbrc.2015.11.050

[18] Choi, L., Vernon, J., Kopach, O., Minett, M.S., Mills, K., Clayton, P.T., Meert, T. and Wood, J.N. (2015) The Fabry disease-associated lipid Lyso-Gb3 enhances voltage-gated calcium currents in sensory neurons and causes pain. Neurosci. Lett., 594, 163–168. 25697597 10.1016/j.neulet.2015.01.084 PMC4411215

[19] Folts, C.J., Scott-Hewitt, N., Proschel, C., Mayer-Proschel, M. and Noble, M. (2016) Lysosomal re-acidification prevents lysosphingolipid-induced lysosomal impairment and cellular toxicity. PLoS Biol., 14, e1002583. 27977664 10.1371/journal.pbio.1002583 PMC5169359

[20] Taguchi A, Ishii S, Mikame M, Maruyama H. (2023) Distinctive accumulation of globotriaosylceramide and globotriaosylsphingosine in a mouse model of classic Fabry disease. Molecular genetics and metabolism reports. 100952, 34. 10.1016/j.ymgmr.2022.100952 PMC9823212 36624895

[21] Aerts, J.M., Groener, J.E., Kuiper, S., Donker-Koopman, W.E., Strijland, A., Ottenhoff, R., van Roomen, C., Mirzaian, M., Wijburg, F.A., Linthorst, G.E. et al. (2008) Elevated globotriaosylsphingosine is a hallmark of Fabry disease. Proc. Natl. Acad. Sci. USA, 105, 2812–2817. 18287059 10.1073/pnas.0712309105 PMC2268542

[22] Rolfs, A., Giese, A.K., Grittner, U., Mascher, D., Elstein, D., Zimran, A., Bottcher, T., Lukas, J., Hubner, R., Golnitz, U. et al. (2013) Glucosylsphingosine is a highly sensitive and specific biomarker for primary diagnostic and follow-up monitoring in Gaucher disease in a non-Jewish, Caucasian cohort of Gaucher disease patients. PLoS One, 8, e79732. 24278166 10.1371/journal.pone.0079732 PMC3835853

[23] Kozlov G, Gehring K. (2020) Calnexin cycle – structural features of the ER chaperone system. FEBS J., 287, 4322–4340. 32285592 10.1111/febs.15330 PMC7687155

[24] Beere, H.M. (2004) ``The stress of dying'': the role of heat shock proteins in the regulation of apoptosis. J. Cell Sci., 117, 2641–2651. 15169835 10.1242/jcs.01284

[25] Lanneau, D., Brunet, M., Frisan, E., Solary, E., Fontenay, M. and Garrido, C. (2008) Heat shock proteins: essential proteins for apoptosis regulation. J. Cell. Mol. Med., 12, 743–761. 18266962 10.1111/j.1582-4934.2008.00273.x PMC4401125

[26] Binder, R.J. (2014) Functions of heat shock proteins in pathways of the innate and adaptive immune system. J. Immunol., 193, 5765–5771. 25480955 10.4049/jimmunol.1401417 PMC4304677

[27] Solis, E.J., Pandey, J.P., Zheng, X., Jin, D.X., Gupta, P.B., Airoldi, E.M., Pincus, D. and Denic, V. (2016) Defining the essential function of yeast Hsf1 reveals a compact transcriptional program for maintaining eukaryotic proteostasis. Mol. Cell, 63, 60–71. 27320198 10.1016/j.molcel.2016.05.014 PMC4938784

[28] Echeverria, P.C., Bernthaler, A., Dupuis, P., Mayer, B. and Picard, D. (2011) An interaction network predicted from public data as a discovery tool: application to the Hsp90 molecular chaperone machine. PLoS One, 6, e26044. 22022502 10.1371/journal.pone.0026044 PMC3195953

[29] Biebl, M.M. and Buchner, J. (2019) Structure, function, and regulation of the Hsp90 machinery. Cold Spring Harb. Perspect. Biol., 11, 1–16. 10.1101/cshperspect.a034017. PMC6719599 30745292

[30] Sanchez-Nino, M.D., Carpio, D., Sanz, A.B., Ruiz-Ortega, M., Mezzano, S. and Ortiz, A. (2015) Lyso-Gb3 activates Notch1 in human podocytes. Hum. Mol. Genet., 24, 5720–5732. 26206887 10.1093/hmg/ddv291

[31] Hofmann, L., Hose, D., Griesshammer, A., Blum, R., Doring, F., Dib-Hajj, S., Waxman, S., Sommer, C., Wischmeyer, E. and Uceyler, N. (2018) Characterization of small fiber pathology in a mouse model of Fabry disease. Elife, 7, e39300. 10.7554/eLife.39300 PMC6255391 30328411

[32] Consolato, F., De Fusco, M., Schaeffer, C., Pieruzzi, F., Scolari, F., Gallieni, M., Lanzani, C., Feriozzi, S. and Rampoldi, L. (2022) alpha-Gal A missense variants associated with Fabry disease can lead to ER stress and induction of the unfolded protein response. Mol. Genet. Metab. Rep., 33, 100926. 36345359 10.1016/j.ymgmr.2022.100926 PMC9636577

[33] Grantham, J. (2020) The molecular chaperone CCT/TRiC: An essential component of proteostasis and a potential modulator of protein aggregation. Front. Genet., 11, 172. 32265978 10.3389/fgene.2020.00172 PMC7096549

[34] Sot, B., Rubio-Munoz, A., Leal-Quintero, A., Martinez-Sabando, J., Marcilla, M., Roodveldt, C. and Valpuesta, J.M. (2017) The chaperonin CCT inhibits assembly of alpha-synuclein amyloid fibrils by a specific, conformation-dependent interaction. Sci. Rep., 7, 40859. 28102321 10.1038/srep40859 PMC5244355

[35] Pavel, M., Imarisio, S., Menzies, F.M., Jimenez-Sanchez, M., Siddiqi, F.H., Wu, X., Renna, M., O'Kane, C.J., Crowther, D.C. and Rubinsztein, D.C. (2016) CCT complex restricts neuropathogenic protein aggregation via autophagy. Nat. Commun., 7, 13821. 27929117 10.1038/ncomms13821 PMC5155164

[36] Chen, X.Q., Fang, F., Florio, J.B., Rockenstein, E., Masliah, E., Mobley, W.C., Rissman, R.A. and Wu, C. (2018) T-complex protein 1-ring complex enhances retrograde axonal transport by modulating tau phosphorylation. Traffic, 19, 840–853. 30120810 10.1111/tra.12610 PMC6191364

[37] Lee, M.J., Stephenson, D.A., Groves, M.J., Sweeney, M.G., Davis, M.B., An, S.F., Houlden, H., Salih, M.A., Timmerman, V., de Jonghe, P. et al. (2003) Hereditary sensory neuropathy is caused by a mutation in the delta subunit of the cytosolic chaperonin-containing t-complex peptide-1 (Cct4 ) gene. Hum. Mol. Genet., 12, 1917–1925. 12874111 10.1093/hmg/ddg198

[38] Bouhouche, A., Benomar, A., Bouslam, N., Chkili, T. and Yahyaoui, M. (2006) Mutation in the epsilon subunit of the cytosolic chaperonin-containing t-complex peptide-1 (Cct5) gene causes autosomal recessive mutilating sensory neuropathy with spastic paraplegia. J. Med. Genet., 43, 441–443. 16399879 10.1136/jmg.2005.039230 PMC2564519

[39] Gomes, A.V., Young, G.W., Wang, Y., Zong, C., Eghbali, M., Drews, O., Lu, H., Stefani, E. and Ping, P. (2009) Contrasting proteome biology and functional heterogeneity of the 20 S proteasome complexes in mammalian tissues. Mol. Cell. Proteomics, 8, 302–315. 18931337 10.1074/mcp.M800058-MCP200 PMC2634581

[40] Shemesh, N., Jubran, J., Dror, S., Simonovsky, E., Basha, O., Argov, C., Hekselman, I., Abu-Qarn, M., Vinogradov, E., Mauer, O. et al. (2021) The landscape of molecular chaperones across human tissues reveals a layered architecture of core and variable chaperones. Nat. Commun., 12, 2180. 33846299 10.1038/s41467-021-22369-9 PMC8042005

[41] Heywood, W.E., Doykov, I., Spiewak, J., Hallqvist, J., Mills, K. and Nowak, A. (2019) Global glycosphingolipid analysis in urine and plasma of female Fabry disease patients. Biochim. Biophys. Acta, 1865, 2726–2735. 10.1016/j.bbadis.2019.07.005 31319156

