# Supplementary material for: Elucidating the toxic effect and disease mechanisms associated with Lyso-Gb3 in Fabry disease
Source: Hum Mol Genet. 2023 May 5;32(15):2464–72. doi: 10.1093/hmg/ddad073 (PMC10360391; doi:10.1093/hmg/ddad073)
Supplement: supplementary_data_revised_ddad073 [file supplementary_data_revised_ddad073.docx]

Elucidating the toxic effect and disease mechanisms associated with Lyso-Gb3 in Fabry disease

Valeria Nikolaenko^1,2^, David G Warnock^3^, *Kevin Mills^1^ and *Wendy E Heywood^1,2^

**Table S1 and S2 – relates to data shown in Figure 2**

**Table S3 relates to data shown in Figure 3**

**Supplementary Data**

**Table S1.** One hundred and thirty seven ubiquitinated proteins and their quantified amount (fmol) unique to the lysoGb3 exposure.

| **Gene** | **Protein** | **Amount (fmol)** |
| --- | --- | --- |
| PTDSS1 | Phosphatidylserine synthase 1 | 8.362 |
| H2BFS | Histone H2B type F-S | 4.082 |
| HIST1H2BA | Histone H2B type 1-A | 2.026 |
| RPS10 | 40S ribosomal protein S10 | 1.627 |
| CANX | Calnexin | 1.621 |
| SRSF3 | Serine/arginine-rich splicing factor 3 | 1.606 |
| VDAC3 | Voltage-dependent anion-selective channel protein 3 | 1.383 |
| HMGB1 | High mobility group protein B1 | 1.351 |
| MT-CO2 | Cytochrome c oxidase subunit 2 | 1.242 |
| HNRNPDL | Heterogeneous nuclear ribonucleoprotein D-like | 1.202 |
| SLC25A6 | ADP/ATP translocase 3 | 1.170 |
| RALY | RNA-binding protein Raly | 1.004 |
| CCT8 | T-complex protein 1 subunit theta | 0.988 |
| HNRNPAB | Heterogeneous nuclear ribonucleoprotein A/B | 0.917 |
| MYL6 | Myosin light polypeptide 6 | 0.888 |
| SRP9 | Signal recognition particle 9 kDa protein | 0.872 |
| NME1 | Nucleoside diphosphate kinase A | 0.826 |
| RBM3 | RNA-binding protein 3 | 0.823 |
| RPL24 | 60S ribosomal protein L24 | 0.796 |
| FUS | RNA-binding protein FUS | 0.793 |
| SSR4 | Translocon-associated protein subunit delta | 0.765 |
| RPS19 | 40S ribosomal protein S19 | 0.751 |
| TMED9 | Transmembrane emp24 domain-containing protein 9 | 0.749 |
| SYNCRIP | Heterogeneous nuclear ribonucleoprotein Q | 0.745 |
| LRRC59 | Leucine-rich repeat-containing protein 59 | 0.735 |
| RAB11A | Ras-related protein Rab-11A | 0.703 |
| RPS14 | 40S ribosomal protein S14 | 0.667 |
| RPL37A | 60S ribosomal protein L37a | 0.662 |
| PABPC1 | Polyadenylate-binding protein 1 | 0.651 |
| ELAVL4 | ELAV-like protein 4 | 0.646 |
| HNRNPR | Heterogeneous nuclear ribonucleoprotein R | 0.635 |
| TUFM | Elongation factor Tu_ mitochondrial | 0.622 |
| RPN1 | Dolichyl-diphosphooligosaccharide--protein glycosyltransferase subunit 1 | 0.597 |
| ALYREF | THO complex subunit 4 | 0.592 |
| ILF3 | Interleukin enhancer-binding factor 3 | 0.586 |
| CIRBP | Cold-inducible RNA-binding protein | 0.537 |
| PAICS | Multifunctional protein ADE2 | 0.500 |
| ATP5PD | ATP synthase subunit d_ mitochondrial | 0.489 |
| CALR | Calreticulin | 0.486 |
| PSMB1 | Proteasome subunit beta type-1 | 0.475 |
| RTN4 | Reticulon-4 | 0.474 |
| SEPTIN9 | Septin-9 | 0.473 |
| PSMB4 | Proteasome subunit beta type-4 | 0.467 |
| COX4I1 | Cytochrome c oxidase subunit 4 isoform 1_ mitochondrial | 0.461 |
| RUVBL1 | RuvB-like 1 | 0.439 |
| ITGB1 | Integrin beta-1 | 0.438 |
| RPL23A | 60S ribosomal protein L23a | 0.436 |
| ATP5ME | ATP synthase subunit e_ mitochondrial | 0.434 |
| NEDD8 | NEDD8 | 0.430 |
| RAB5C | Ras-related protein Rab-5C | 0.428 |
| DDX39A | ATP-dependent RNA helicase DDX39A | 0.422 |
| RPN2 | Dolichyl-diphosphooligosaccharide--protein glycosyltransferase subunit 2 | 0.420 |
| PSMA2 | Proteasome subunit alpha type-2 | 0.411 |
| XRCC5 | X-ray repair cross-complementing protein 5 | 0.407 |
| PSMA4 | Proteasome subunit alpha type-4 | 0.401 |
| HSPE1 | 10 kDa heat shock protein_ mitochondrial | 0.382 |
| CDC42 | Cell division control protein 42 homolog | 0.380 |
| DLD | Dihydrolipoyl dehydrogenase_ mitochondrial | 0.375 |
| FH | Fumarate hydratase_ mitochondrial | 0.373 |
| RPLP1 | 60S acidic ribosomal protein P1 | 0.362 |
| ARL3 | ADP-ribosylation factor-like protein 3 | 0.360 |
| PSMC5 | 26S proteasome regulatory subunit 8 | 0.359 |
| PSMB2 | Proteasome subunit beta type-2 | 0.354 |
| HSD17B4 | Peroxisomal multifunctional enzyme type 2 | 0.338 |
| SNRPB2 | U2 small nuclear ribonucleoprotein B'' | 0.338 |
| ERH | Enhancer of rudimentary homolog | 0.333 |
| TRA2A | Transformer-2 protein homolog alpha | 0.329 |
| ATP5PB | ATP synthase F(0) complex subunit B1_ mitochondrial | 0.326 |
| RAB2A | Ras-related protein Rab-2A | 0.326 |
| SLC25A13 | Calcium-binding mitochondrial carrier protein Aralar2 | 0.322 |
| SEPTIN6 | Septin-6 | 0.314 |
| HSPA4L | Heat shock 70 kDa protein 4L | 0.311 |
| STOML2 | Stomatin-like protein 2_ mitochondrial | 0.306 |
| RAB35 | Ras-related protein Rab-35 | 0.297 |
| ATP6V0C | V-type proton ATPase 16 kDa proteolipid subunit | 0.290 |
| DNAJA1 | DnaJ homolog subfamily A member 1 | 0.288 |
| TMPO | Lamina-associated polypeptide 2_ isoforms beta/gamma | 0.285 |
| HADH | Hydroxyacyl-coenzyme A dehydrogenase_ mitochondrial | 0.285 |
| TMEM167A | Protein kish-A | 0.280 |
| IMMT | MICOS complex subunit MIC60 | 0.277 |
| ARPC3 | Actin-related protein 2/3 complex subunit 3 | 0.272 |
| TMEM14C | Transmembrane protein 14C | 0.264 |
| PA2G4 | Proliferation-associated protein 2G4 | 0.257 |
| EIF3B | Eukaryotic translation initiation factor 3 subunit B | 0.254 |
| ALCAM | CD166 antigen | 0.248 |
| NIPSNAP1 | Protein NipSnap homolog 1 | 0.245 |
| RNPS1 | RNA-binding protein with serine-rich domain 1 | 0.244 |
| HSD17B12 | Very-long-chain 3-oxoacyl-CoA reductase | 0.239 |
| RPS26 | 40S ribosomal protein S26 | 0.236 |
| DES | Desmin | 0.236 |
| COX6A1 | Cytochrome c oxidase subunit 6A1_ mitochondrial | 0.233 |
| SSBP1 | Single-stranded DNA-binding protein_ mitochondrial | 0.232 |
| PRDX3 | Thioredoxin-dependent peroxide reductase_ mitochondrial | 0.223 |
| CDK1 | Cyclin-dependent kinase 1 | 0.221 |
| SLC25A11 | Mitochondrial 2-oxoglutarate/malate carrier protein | 0.218 |
| FLNA | Filamin-A | 0.216 |
| SRSF9 | Serine/arginine-rich splicing factor 9 | 0.215 |
| SSB | Lupus La protein | 0.215 |
| SF3B6 | Splicing factor 3B subunit 6 | 0.214 |
| TPM3 | Tropomyosin alpha-3 chain | 0.208 |
| PRMT1 | Protein arginine N-methyltransferase 1 | 0.207 |
| RP1BL | Ras-related protein Rap-1b-like protein | 0.200 |
| SAR1A | GTP-binding protein SAR1a | 0.196 |
| MCM4 | DNA replication licensing factor MCM4 | 0.195 |
| SRSF2 | Serine/arginine-rich splicing factor 2 | 0.195 |
| RPL28 | 60S ribosomal protein L28 | 0.194 |
| COX7C | Cytochrome c oxidase subunit 7C_ mitochondrial | 0.191 |
| SEC61A1 | Protein transport protein Sec61 subunit alpha isoform 1 | 0.189 |
| RAB6B | Ras-related protein Rab-6B | 0.189 |
| EIF3I | Eukaryotic translation initiation factor 3 subunit I | 0.188 |
| YKT6 | Synaptobrevin homolog YKT6 | 0.182 |
| MCM2 | DNA replication licensing factor MCM2 | 0.173 |
| SNU13 | NHP2-like protein 1 | 0.170 |
| ACAT1 | Acetyl-CoA acetyltransferase_ mitochondrial | 0.160 |
| PAFAH1B3 | Platelet-activating factor acetylhydrolase IB subunit gamma | 0.155 |
| PSMC4 | 26S proteasome regulatory subunit 6B | 0.148 |
| PSMC3 | 26S proteasome regulatory subunit 6A | 0.147 |
| COX7A2 | Cytochrome c oxidase subunit 7A2_ mitochondrial | 0.139 |
| UBQLN4 | Ubiquilin-4 | 0.138 |
| CLIC6 | Chloride intracellular channel protein 6 | 0.132 |
| LYPLA2 | Acyl-protein thioesterase 2 | 0.131 |
| GNAI1 | Guanine nucleotide-binding protein G(i) subunit alpha-1 | 0.113 |
| PSMD3 | 26S proteasome non-ATPase regulatory subunit 3 | 0.110 |
| UBQLN1 | Ubiquilin-1 | 0.108 |
| PRXL2A | Peroxiredoxin-like 2A | 0.102 |
| KRT7 | Keratin_ type II cytoskeletal 7 | 0.100 |
| H2AFX | Histone H2AX | 0.089 |
| NACA | Nascent polypeptide-associated complex subunit alpha_ muscle-specific form | 0.086 |
| ACTN3 | Alpha-actinin-3 | 0.083 |
| NEFM | Neurofilament medium polypeptide | 0.065 |
| PTBP2 | Polypyrimidine tract-binding protein 2 | 0.057 |
| LDHAL6B | L-lactate dehydrogenase A-like 6B | 0.037 |
| TUBB8 | Tubulin beta-8 chain | 0.026 |
| Total |  | 70.184 |

**Table S2.** The ninety-nine ubiquitinated proteins and their quantified amounts (fmol) commonly identified in control, GlcSph and LysoGb3 datasets.

|  |  | **Amount (fmol)** | | |
| --- | --- | --- | --- | --- |
| **Gene** | **Protein** | **Control** | **GlcSph** | **LysoGb3** |
| HIST1H4A | Histone H4 | 5.5511 | 12.1768 | 16.6589 |
| PARK7 | Protein/nucleic acid deglycase DJ-1 | 5.2933 | 10.6715 | 10.5507 |
| TPI1 | Triosephosphate isomerase | 3.4112 | 7.2767 | 8.601 |
| GAPDH | Glyceraldehyde-3-phosphate dehydrogenase | 3.1344 | 7.0635 | 14.0963 |
| KRT1 | Keratin_ type II cytoskeletal 1 | 2.9801 | 6.9027 | 3.4204 |
| IGHG1 | Immunoglobulin heavy constant gamma 1 | 2.4398 | 1.8026 | 1.6132 |
| TUBA1B | Tubulin alpha-1B chain | 2.3541 | 9.4854 | 2.1672 |
| EEF1A2 | Elongation factor 1-alpha 2 | 1.9958 | 1.9735 | 6.7502 |
| KRT9 | Keratin_ type I cytoskeletal 9 | 1.6397 | 3.2229 | 0.9802 |
| FABP4 | Fatty acid-binding protein_ adipocyte | 1.5336 | 3.6646 | 3.1735 |
| HSPD1 | 60 kDa heat shock protein_ mitochondrial | 1.4285 | 1.5481 | 4.6047 |
| TUBB | Tubulin beta chain | 1.3234 | 7.3832 | 9.0997 |
| KRT10 | Keratin_ type I cytoskeletal 10 | 1.1571 | 3.8456 | 1.5936 |
| PEBP1 | Phosphatidylethanolamine-binding protein 1 | 1.1259 | 3.2847 | 2.3055 |
| HIST1H3A | Histone H3.1 | 1.0605 | 1.9787 | 4.0135 |
| HIST2H3A | Histone H3.2 | 0.9732 | 3.0158 | 6.7745 |
| ATP5F1A | ATP synthase subunit alpha_ mitochondrial | 0.8981 | 1.1029 | 3.9487 |
| ALB | Serum albumin | 0.888 | 1.4738 | 2.0343 |
| HNRPK | Heterogeneous nuclear ribonucleoprotein K | 0.8869 | 2.0649 | 5.0235 |
| PFN1 | Profilin-1 | 0.8815 | 2.3404 | 3.8053 |
| HIST1H2B1O | Histone H2B type 1-O | 0.8413 | 1.8933 | 1.1453 |
| HS90AB1 | Heat shock protein HSP 90-beta | 0.8344 | 3.2493 | 5.8623 |
| PKM | Pyruvate kinase PKM | 0.805 | 3.1385 | 4.6 |
| MDH1 | Malate dehydrogenase_ cytoplasmic | 0.7887 | 1.2138 | 1.6317 |
| ATP5F1B | ATP synthase subunit beta_ mitochondrial | 0.7846 | 1.5847 | 2.9478 |
| H2AFV | Histone H2A.V | 0.7568 | 0.8014 | 2.446 |
| IGKC | Immunoglobulin kappa constant | 0.7541 | 0.5088 | 0.5057 |
| MIF | Macrophage migration inhibitory factor | 0.7478 | 2.4199 | 5.3933 |
| HSPA1B | Heat shock cognate 71 kDa protein | 0.7183 | 3.9156 | 4.2852 |
| PPIA | Peptidyl-prolyl cis-trans isomerase A | 0.7054 | 3.4986 | 6.8876 |
| HS90AA1 | Heat shock protein HSP 90-alpha | 0.6667 | 2.1141 | 4.1479 |
| EIF4A1 | Eukaryotic initiation factor 4A-I | 0.6626 | 1.2083 | 2.6946 |
| LDHB | L-lactate dehydrogenase B chain | 0.611 | 1.9757 | 3.8908 |
| HNRPC | Heterogeneous nuclear ribonucleoproteins C1/C2 | 0.6013 | 1.7603 | 5.5274 |
| FABP5 | Fatty acid-binding protein 5 | 0.5966 | 1.8709 | 1.1354 |
| DPYSL3 | Dihydropyrimidinase-related protein 3 | 0.5856 | 0.8677 | 1.7629 |
| MDH2 | Malate dehydrogenase_ mitochondrial | 0.5775 | 1.6845 | 1.8071 |
| HNRNPA2B1 | Heterogeneous nuclear ribonucleoproteins A2/B1 | 0.5756 | 3.0153 | 8.7724 |
| HSD17B10 | 3-hydroxyacyl-CoA dehydrogenase type-2 | 0.5672 | 0.6069 | 1.9384 |
| HIST1H1B | Histone H1.5 | 0.5403 | 0.9041 | 2.1103 |
| FABP3 | Fatty acid-binding protein_ heart | 0.5206 | 1.8389 | 1.3016 |
| CCT6A | T-complex protein 1 subunit zeta | 0.5112 | 0.3699 | 1.7897 |
| CCT3 | T-complex protein 1 subunit gamma | 0.4909 | 0.4017 | 0.9698 |
| RPS3 | 40S ribosomal protein S3 | 0.4857 | 1.3577 | 3.8741 |
| RPS20 | 40S ribosomal protein S20 | 0.4819 | 0.6363 | 1.6169 |
| TCP1 | T-complex protein 1 subunit alpha | 0.4507 | 0.3224 | 1.232 |
| YWHAE | 14-3-3 protein epsilon | 0.45 | 1.4997 | 2.9046 |
| ACTC1 | Actin_ alpha cardiac muscle 1 | 0.443 | 0.9898 | 1.6286 |
| KRT2 | Keratin_ type II cytoskeletal 2 epidermal | 0.4187 | 1.4371 | 0.357 |
| HSPA5 | Endoplasmic reticulum chaperone BiP | 0.4123 | 1.0112 | 2.1888 |
| HIST2H2BD | Putative histone H2B type 2-D | 0.4091 | 0.7098 | 2.8858 |
| CFL2 | Cofilin-2 | 0.4068 | 0.546 | 0.4522 |
| ENO1 | Alpha-enolase | 0.389 | 3.6323 | 3.2379 |
| CKB | Creatine kinase B-type | 0.3879 | 0.9016 | 1.5189 |
| H3F3A | Histone H3.3 | 0.3732 | 0.7906 | 3.2517 |
| RAN | GTP-binding nuclear protein Ran | 0.3692 | 1.4279 | 2.7387 |
| CCT7 | T-complex protein 1 subunit eta | 0.355 | 0.3949 | 0.7529 |
| VIM | Vimentin | 0.3351 | 4.4355 | 14.4521 |
| CFL1 | Cofilin-1 | 0.3232 | 2.4226 | 3.9194 |
| LDHA | L-lactate dehydrogenase A chain | 0.3066 | 1.3639 | 1.9839 |
| HNRPM | Heterogeneous nuclear ribonucleoprotein M | 0.2915 | 0.5252 | 2.2512 |
| YWHAZ | 14-3-3 protein zeta/delta | 0.2813 | 1.1152 | 1.8219 |
| UCHL1 | Ubiquitin carboxyl-terminal hydrolase isozyme L1 | 0.2804 | 1.0225 | 2.5136 |
| NPM1 | Nucleophosmin | 0.2688 | 0.7362 | 2.159 |
| CCT4 | T-complex protein 1 subunit delta | 0.2588 | 0.2509 | 0.6394 |
| HSPB1 | Heat shock protein beta-1 | 0.2518 | 1.6338 | 2.3268 |
| TUBB3 | Tubulin beta-3 chain | 0.2502 | 1.8574 | 1.5506 |
| LGALS1 | Galectin-1 | 0.2483 | 1.0276 | 1.7763 |
| RPL11 | 60S ribosomal protein L11 | 0.2474 | 0.8304 | 1.8934 |
| SERPINH1 | Serpin H1 | 0.2453 | 0.3511 | 2.7581 |
| HSP90B1 | Endoplasmin | 0.2419 | 0.7847 | 2.4719 |
| NONO | Non-POU domain-containing octamer-binding protein | 0.2408 | 0.3887 | 2.3567 |
| CCT2 | T-complex protein 1 subunit beta | 0.238 | 0.2913 | 1.6972 |
| RPL12 | 60S ribosomal protein L12 | 0.2357 | 0.1639 | 1.7281 |
| HNRPD | Heterogeneous nuclear ribonucleoprotein D0 | 0.2209 | 0.4669 | 2.6573 |
| YWHAQ | 14-3-3 protein theta | 0.2098 | 0.5247 | 0.9498 |
| UBE2D3 | Ubiquitin-conjugating enzyme E2 D3 | 0.2096 | 0.4502 | 0.6871 |
| PPIB | Peptidyl-prolyl cis-trans isomerase B | 0.2087 | 0.7349 | 3.3304 |
| TUBB2B | Tubulin beta-2B chain | 0.1954 | 1.7357 | 1.969 |
| PTBP1 | Polypyrimidine tract-binding protein 1 | 0.1931 | 0.6909 | 3.409 |
| RPL30 | 60S ribosomal protein L30 | 0.1915 | 1.4212 | 2.4863 |
| DPYSL2 | Dihydropyrimidinase-related protein 2 | 0.1866 | 0.2186 | 0.8785 |
| ILF2 | Interleukin enhancer-binding factor 2 | 0.1758 | 0.1978 | 1.9227 |
| TUBB6 | Tubulin beta-6 chain | 0.1498 | 0.5654 | 0.6641 |
| PRDX1 | Peroxiredoxin-1 | 0.1467 | 0.4507 | 0.9116 |
| RPSA | 40S ribosomal protein SA | 0.1424 | 0.9522 | 1.4188 |
| HNRH1 | Heterogeneous nuclear ribonucleoprotein H | 0.1335 | 0.465 | 2.479 |
| EEF1G | Elongation factor 1-gamma | 0.1309 | 0.5761 | 0.7725 |
| BANF1 | Barrier-to-autointegration factor | 0.1208 | 0.2572 | 1.543 |
| SFPQ | Splicing factor_ proline- and glutamine-rich | 0.1201 | 0.3068 | 2.0067 |
| TUBB4B | Tubulin beta-4B chain | 0.1141 | 1.5627 | 2.0384 |
| PCNA | Proliferating cell nuclear antigen | 0.1136 | 0.8057 | 0.9816 |
| RPL7A | 60S ribosomal protein L7a | 0.1117 | 0.4219 | 0.944 |
| HNRH2 | Heterogeneous nuclear ribonucleoprotein H2 | 0.1003 | 0.0587 | 0.3115 |
| RPS7 | 40S ribosomal protein S7 | 0.0967 | 0.5249 | 1.2906 |
| CRMP1 | Dihydropyrimidinase-related protein 1 | 0.077 | 0.1483 | 7.946 |
| HS71B | Heat shock 70 kDa protein 1B | 0.0702 | 0.4794 | 0.6215 |
| PSMB5 | Proteasome subunit beta type-5 | 0.0681 | 0.0967 | 0.3677 |
| HNRNPA1 | Heterogeneous nuclear ribonucleoprotein A1 | 0 | 2.1246 | 8.4616 |
| Total amount |  | 70.0646 | 184.2159 | 312.7565 |

**Table S3.** Proteins identified in duplicate analyses of lyso-Gb3 binding/interactor analyses. Non-specific binding proteins identified in blank bead analyses were removed. Proteins also found to bind to glucosylshingosine are indicated in bold.

| Accession | Description | |
| --- | --- | --- |
| O60814 | **Histone H2B type 1-K** | |
| O75608 | **Acyl-protein thioesterase 1** | |
| P05141 | **ADP/ATP translocase 2** | |
| P07195 | **L-lactate dehydrogenase B chain** | |
| P12236 | **ADP/ATP translocase 3** | |
| P14406 | Cytochrome c oxidase subunit 7A2_ mitochondrial | |
| P35232 | **Prohibitin** | |
| P40939 | **Trifunctional enzyme subunit alpha_ mitochondrial** | |
| P60709 | **Actin_ cytoplasmic 1** | |
| Q00839 | **Heterogeneous nuclear ribonucleoprotein U** | |
| Q15365 | **Poly(rC)-binding protein 1** | |
| Q15366 | **Poly(rC)-binding protein 2** | |
| Q99714 | **3-hydroxyacyl-CoA dehydrogenase type-2** | |
| O95336 | 6-phosphogluconolactonase | |
| P10809 | 60 kDa heat shock protein_ mitochondrial | |
| P0DMV8 | Heat shock 70 kDa protein 1A | |
| P07900 | Heat shock protein HSP 90-alpha | |
| P08238 | Heat shock protein HSP 90-beta | |
| P00338 | L-lactate dehydrogenase A chain | |
| P25787 | Proteasome subunit alpha type-2 | |
| Q99460 | 26S proteasome non-ATPase regulatory subunit 1 | |
| P53396 | ATP-citrate synthase | |
| O96019 | Actin-like protein 6A | |
| P11766 | Alcohol dehydrogenase class-3 | |
| P61163 | Alpha-centractin | |
| Q00610 | Clathrin heavy chain 1 | |
| O75367 | Core histone macro-H2A.1 | |
| Q9BPU6 | Dihydropyrimidinase-related protein 5 | |
| P49736 | DNA replication licensing factor MCM2 | |
| P13639 | Elongation factor 2 | |
| Q16658 | Fascin |  |
| P30043 | Flavin reductase (NADPH) | |
| P22626 | Heterogeneous nuclear ribonucleoproteins A2/B1 | |
| P04908 | Histone H2A type 1-B/E | |
| O95373 | Importin-7 | |
| Q12905 | Interleukin enhancer-binding factor 2 | |
| P43243 | Matrin-3 |  |
| Q16891 | MICOS complex subunit MIC60 | |
| P26599 | Polypyrimidine tract-binding protein 1 | |
| Q9UHG3 | Prenylcysteine oxidase 1 | |
| P61619 | Protein transport protein Sec61 subunit alpha isoform 1 | |
| Q99497 | Protein/nucleic acid deglycase DJ-1 | |
| Q13283 | Ras GTPase-activating protein-binding protein 1 | |
| Q9UBT2 | SUMO-activating enzyme subunit 2 | |
| Q99536 | Synaptic vesicle membrane protein VAT-1 homolog | |
| P17987 | T-complex protein 1 subunit alpha | |
| P50991 | T-complex protein 1 subunit delta | |
| P40227 | T-complex protein 1 subunit zeta | |
| Q15631 | Translin |  |
| P07437 | Tubulin beta chain | |
| Q13509 | Tubulin beta-3 chain | |
| P22314 | Ubiquitin-like modifier-activating enzyme 1 | |
